# Virtual Reality–Based Relaxation Training and Symptom Improvement Among Inpatients With Depressive Disorders: Retrospective Nonrandomized Comparative Study

**DOI:** 10.2196/75251

**Published:** 2026-07-03

**Authors:** Linhui Liu, Wenqian Zhao, Mengqing He, Xue Yang, Kunqiang Yu, Ningning Ding, Wenwen Tian, Gaoyang Liu, Guohua Zhang, Yan Zhang

**Affiliations:** 1 Lishui Second People’s Hospital Affiliated to Wenzhou Medical University, Lishui, China; 2 Department of Psychology, School of Mental Health, Wenzhou Medical University, Chashan Higher Education Park, Wenzhou, Zhejiang, 325035, China, 86 0577-86699862; 3 Cixi Biomedical Research Institute, Wenzhou Medical University, Ningbo, China; 4 JC School of Public Health and Primary Care, Faculty of Medicine, The Chinese University of Hong Kong, Hong Kong, China (Hong Kong); 5 Zhejiang Provincial Clinical Research Center for Mental Disorders, The Affiliated Kangning Hospital of Wenzhou Medical University, Wenzhou, China; 6 Wenzhou Key Laboratory of Basic and Translational Research for Mental Disorders, Wenzhou, China; 7 See Acknowledgments

**Keywords:** virtual reality, VR, relaxation training, depression, anxiety, inpatients

## Abstract

**Background:**

Virtual reality (VR) is increasingly used for adjunctive relaxation training in psychiatric care. However, evidence remains limited among hospitalized patients with depressive disorders, particularly in routine inpatient settings in China, and little is known about whether improvement varies by session frequency.

**Objective:**

This retrospective study examined whether adjunctive VR-based relaxation training was associated with changes in depressive and anxiety symptoms among inpatients with depressive disorders and whether improvement differed by session frequency.

**Methods:**

We conducted a retrospective, nonrandomized natural-group comparison using complete anonymized medical records from patients hospitalized in Lishui Second People’s Hospital between January 1 and December 31, 2022. Patients met *International Classification of Diseases, Tenth Revision* (*ICD-10*) diagnostic criteria for depressive episodes or recurrent depressive disorders and were screened using predefined criteria. The analytic sample included 133 inpatients: 63 (47.4%) received adjunctive VR-based relaxation training plus usual care and 70 (52.6%) received usual care only. Usual care included pharmacotherapy and physiotherapy. The VR intervention consisted of 25-minute immersive relaxation sessions delivered approximately 3 times per week. Symptoms were assessed at admission and discharge using the 17-item Hamilton Depression Scale and Hamilton Anxiety Rating Scale. Response was defined as a reduction of 50% or more from baseline, and remission was defined as a total score of 7 or less. Baseline characteristics, outcome scores, response and remission rates, and exploratory session-frequency subgroups were compared. All analyzed variables were checked against complete medical records; no missing values were identified, and no imputation was performed.

**Results:**

The VR and control groups did not differ significantly in baseline depressive or anxiety scores. At discharge, adjunctive VR-based relaxation training was associated with lower depressive and anxiety symptom scores than usual care alone. The VR group also showed higher response rates for both depressive and anxiety symptoms and a higher anxiety remission rate, whereas depression remission was similar. Exploratory session-frequency analyses suggested that anxiety improvement may be more consistently associated with VR exposure than depression remission; however, the pattern was not strictly linear and should be interpreted cautiously because treatment frequency was linked to hospitalization duration and routine care factors.

**Conclusions:**

This study is innovative in evaluating structured VR-based relaxation training as an adjunct to routine inpatient depression care and in providing preliminary observations on session-frequency patterns in a real-world Chinese psychiatric setting. Unlike many previous VR studies conducted in noninpatient, nonclinical, or short-term experimental contexts, this study reflects everyday clinical practice among hospitalized patients with depressive disorders. The findings contribute practical evidence for integrating immersive relaxation into comprehensive inpatient care, particularly when additional anxiety relief is desired. Because the study was retrospective and nonrandomized, the findings indicate associations rather than causal effects and should be confirmed in prospective randomized controlled trials.

## Introduction

Depression is a common chronic disease and significant public health issue. In 2008, the World Health Organization (WHO) ranked major depression as the third cause of burden of disease worldwide and projected that the disease would become the top one by 2030 [1]. In China, the 12-month prevalence of depression is 3.6% and the lifetime prevalence is 6.8%; about 95 million people in China have depression [2]. From 1999 to 2017, depression rose from 15th to the 10th leading cause of disability-adjusted life years in China [3]. Patients diagnosed with major depressive disorder (MDD) typically exhibit 2 core categories of symptoms: depressive symptoms (eg, depressed mood and anhedonia) and anxiety symptoms (eg, excessive worry and somatic tension). It is noteworthy that approximately 45% to 75% of patients diagnosed with MDD also exhibit substantial anxiety symptoms or comorbid anxiety disorders [4]. Such comorbid patients tend to exhibit more severe functional impairment and a higher risk of suicide [5-7]. Consequently, the synchronized treatment of anxiety symptoms constitutes a pivotal element of clinical intervention in MDD and cannot be disregarded. Four broad categories of interventions are currently available for depression treatment—generic psychosocial interventions, formulation-based interventions of psychological therapy, pharmacotherapy, and electroconvulsive therapy [8]. Pharmacotherapy is less preferred as medication may have side effects for patients [9]. However, in low-resource areas such as China, mental health services and professionals are lacking, and individuals with depression are not treated in a timely and adequate manner. A survey showed that only 9.5% of patients with depressive disorders received mental health services (eg, psychiatric-psychological specialist treatment and integrated unit treatment) [10]. Innovative mental health treatments that improve both access to care and treatment efficacy are warranted.

In recent years, emerging research has applied virtual reality (VR) technology to facilitate depression treatment. It has been demonstrated that VR relaxation training exerts a modulatory effect on the stress response system through multiple physiological pathways. First, the core mechanism of action of the aforementioned treatment involves the activation of the parasympathetic nervous system, which is a key indicator of the body’s ability to achieve deep relaxation and stress recovery. Research has demonstrated that VR-based training in positive thinking meditation significantly enhances parameters of parasympathetic activity [11]. Second, at the neuroendocrine level, intervention experiments with locomotive crew demonstrated that VR relaxation training reduced serum cortisol levels from 6.75 to 6.28 ng/mL [12], confirming its inhibitory effect on the hypothalamo-pituitary-adrenal axis of stress response. Furthermore, immersive VR environments comprising natural scenes have been demonstrated to enhance heart rate variability and promote enhanced autonomic coordination by means of augmenting cardiac vagal tone [13]. Jingili et al [14] used bibliometric analyses and found a growing trend in the use of VR for depression and anxiety from 1995 to 2022 (n=1897). This systematic review demonstrated that VR interventions are more effective than control (ie, usual care) in reducing anxiety, depression, fatigue, and pain. VR has been shown to effectively reduce these symptoms in patients with diverse conditions, including cancer [15]. For example, a randomized controlled trial of older women found that an immersive VR program integrating relaxation and psychotherapeutic elements reduced depression-anxiety scores more significantly than traditional group relaxation [16]. However, barriers exist including difficulty in keeping patients engaged and focused [17]. VR-based relaxation training can help patients to immerse themselves in the relaxation training situation and mobilize multisensory involvement, which may enhance observed symptom improvements. However, most VR for depression and anxiety studies were conducted in Western cultures, and few studies have been found in Chinese populations [18]. Therefore, it is necessary to further investigate the observed symptom improvement of VR-based relaxation training on depressive and anxiety symptoms in Chinese patients with depression.

On the basis of the findings of previous studies, this study hypothesized that VR-based relaxation training may be associated with improvements in depressive and anxiety symptoms in Chinese patients with depression and attempted to further explore the potential clinically useful session range of VR therapy.

## Methods

### Conditions and Design

This retrospective, nonrandomized, natural-group comparison used anonymized medical records from inpatients with depressive disorders who were treated in the Clinical Psychology Department of Lishui Second People’s Hospital, Lishui, China, from January 1 to December 31, 2022. The VR group was naturally defined by receipt of adjunctive VR-based relaxation training during hospitalization, whereas the control group received usual care only. Because group allocation was not randomized and was based on routine clinical care, the study can identify associations but cannot establish causal effects.

### Inclusion and Exclusion

Patients were eligible if they met *International Classification of Diseases, Tenth Revision* (*ICD-10*) diagnostic criteria for depressive episodes or recurrent depressive disorders, were aged 16 to 60 years, and did not receive psychotherapy or modified electroconvulsive therapy during hospitalization. Patients were excluded if they had severe comorbidities; significant visual or auditory deficits; a history of psychotic features, psychotic disorders, or affective disorders; high suicide risk (Mini International Neuropsychiatric Interview suicidal tendency item score ≥10); severe personality disorders (eg, paranoid or antisocial personality disorder); or intellectual disability. Patients with other psychiatric comorbidities were not excluded.

### Participant Characteristics

A total of 665 inpatient records were screened. After applying inclusion and exclusion criteria, 133 (20%) patients were included in the analytic sample (including n=63, 47.4% in the VR group and n=70, 52.6% in the control group). Major demographic and clinical characteristics included age, sex, education, marital status, occupation, BMI, course of disease, length of hospitalization, family history, number of repetitive transcranial magnetic stimulation (rTMS) sessions, and number of biofeedback sessions.

### Sampling Procedures

All available inpatient medical records from the predefined study period were screened. The study therefore used a retrospective consecutive-record sampling approach rather than prospective recruitment. Data were collected from hospital records and clinician-rated admission and discharge assessments. The setting was a single psychiatric hospital department. No new participants were recruited for this retrospective analysis, and no compensation was provided.

### Sample Size, Power, and Precision

The sample size was determined by the number of eligible complete medical records available during the study period. Because this was a retrospective analysis of routine clinical data, no a priori power calculation was conducted before data collection. To describe the precision of the estimates, we reported 95% CIs for mean differences and odds ratios (ORs) wherever applicable.

### Measures and Covariates

The primary symptom measures were the 17-item Hamilton Depression Rating Scale (HAMD) [19] and the Hamilton Anxiety Rating Scale (HAMA) [20], assessed at admission and discharge. Response was defined as a 50% or greater reduction from baseline in HAMD or HAMA total score, and remission was defined as a total score ≤7 [21]. Demographic and clinical covariates used for baseline comparability analyses included age, sex, education, marital status, occupation, BMI, course of disease, length of hospitalization, family history, rTMS sessions, and biofeedback sessions.

### Quality of Measurements

HAMD and HAMA ratings were conducted by trained attending physicians using standardized interviews. Assessors received standardized training on item interpretation, scoring criteria, interview procedures, and interrater reliability. Assessments were conducted in quiet, private rooms at admission and discharge.

### Treatment Conditions

**Figure 1.**
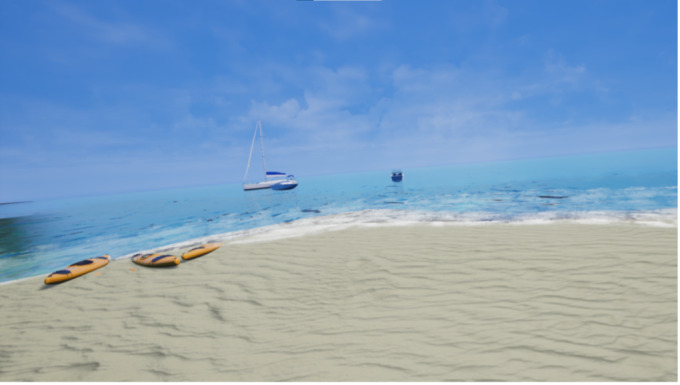
Relaxation training scene 1: sea and sky merge in one color.

**Figure 2.**
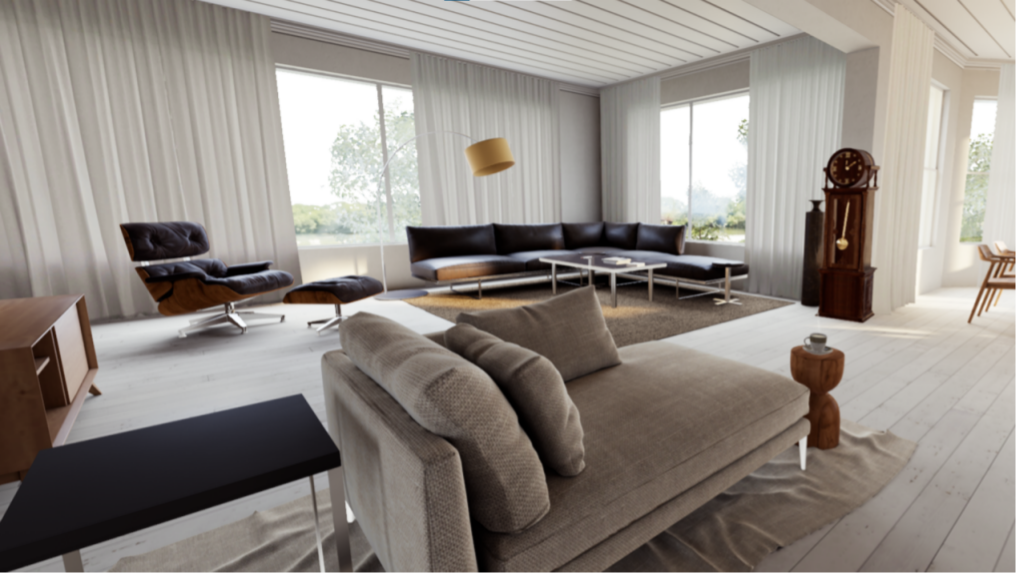
Relaxation training scene 2: comfortable room.

**Figure 3.**
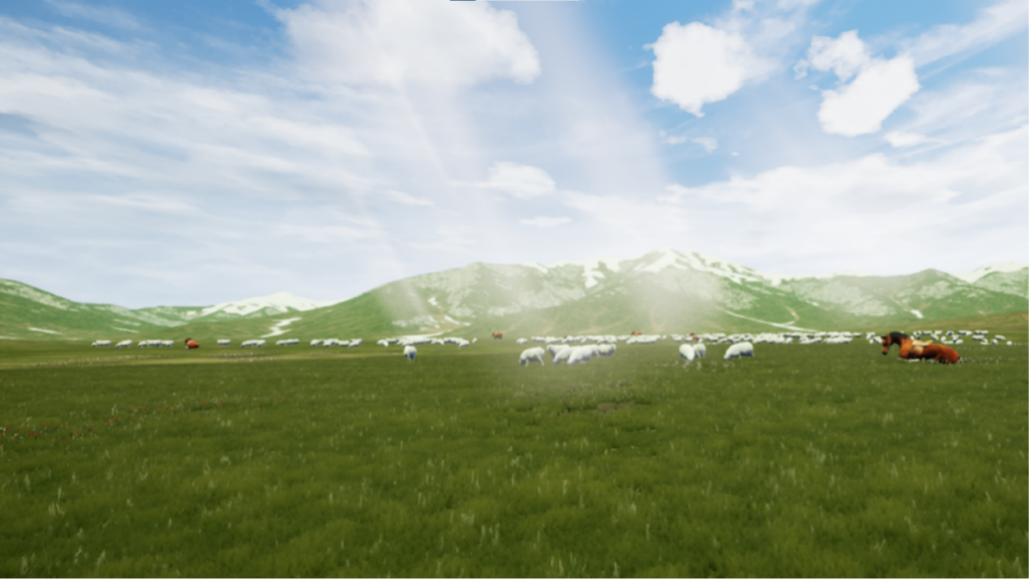
Relaxation training scene 3: grassland scenery.

**Figure 4.**
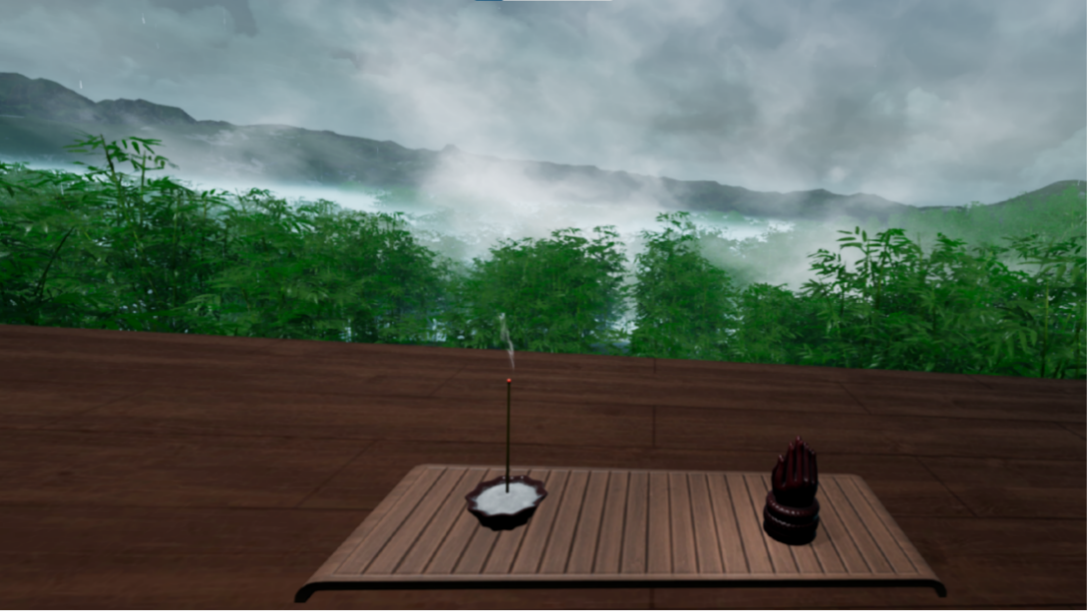
Relaxation training scene 4: forest scenery.

**Figure 5.**
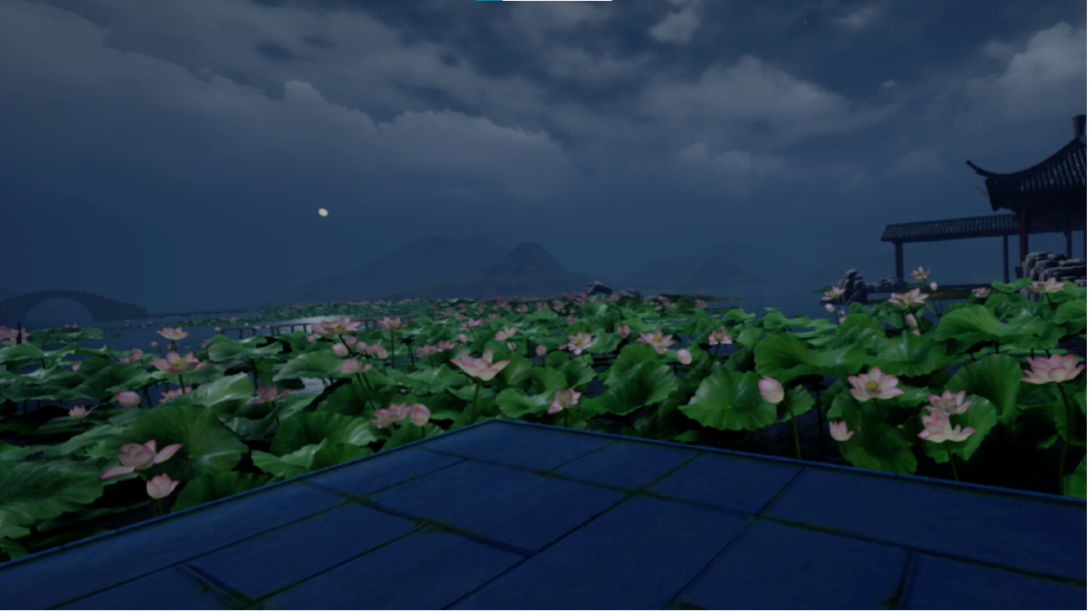
Relaxation training scene 5: moonlight in a lotus pond.

**Figure 6.**
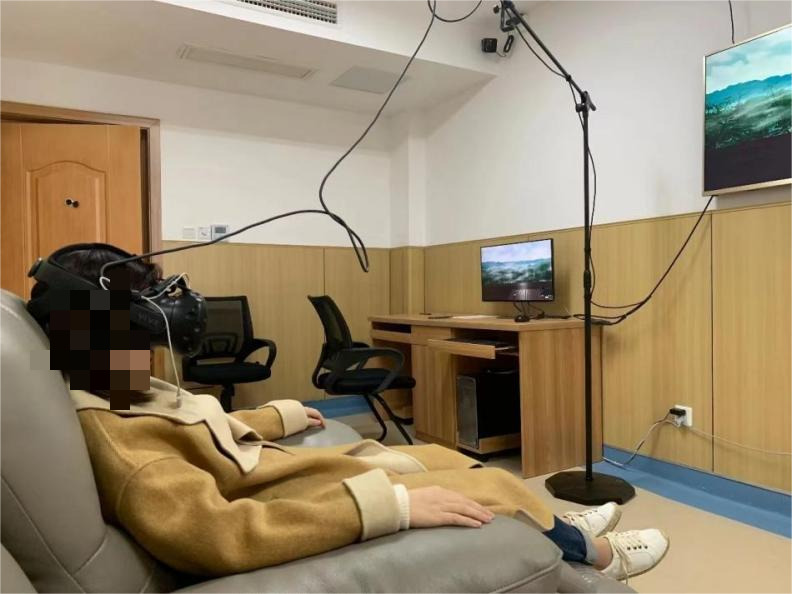
Schematic diagram of the experimental scene. The individual shown in the figure has been masked and deidentified.

All patients received usual care, including antidepressant medication and physiotherapy as clinically indicated. Physiotherapy included rTMS and biofeedback therapy. Patients in the VR group additionally received 25-minute VR-based relaxation training approximately 3 times per week. The VR program used immersive natural or indoor relaxation scenes with audio guidance based on progressive relaxation and/or autogenic techniques. All VR sessions were delivered by the same trained psychotherapist. Representative VR relaxation training scenes are shown in Figures 1-5, and the experimental setting is shown in Figure 6.

### Data Collection and Masking

Baseline assessments were conducted at admission, and posttreatment assessments were conducted at discharge. Data were extracted from complete anonymized medical records. Because this was a retrospective naturalistic study conducted in a routine clinical setting, prospective masking of patients and clinicians to treatment exposure was not feasible. However, symptom assessments were conducted using standardized clinician-rated scales and predefined scoring criteria to reduce assessment variability.

### Data Diagnostics and Missing Data

Before analysis, all variables included in the analytic dataset were checked against the complete medical records. No missing values were identified for any variables included in the analyses; therefore, no data imputation was performed. Distributional characteristics were evaluated to determine the appropriate statistical tests for continuous variables.

### Statistical Analyses

SPSS (version 24; IBM Corp) was used for statistical analyses. Baseline demographic and clinical variables were compared between groups using independent samples *t* tests for normally distributed continuous variables, Mann-Whitney *U* tests for nonnormally distributed continuous variables, and chi-square tests or Fisher exact tests for categorical variables as appropriate. Between-group differences in HAMD and HAMA scores were evaluated using mean differences and 95% CIs. Response and remission rates were compared using ORs and 95% CIs. Exploratory analyses examined session-frequency subgroups within the VR group and hospitalization duration–matched control subgroups. All tests were 2-sided, with statistical significance set at *P*<.05.

### Ethical Considerations

This study involved a retrospective secondary analysis of human-subject medical record data and was reviewed and approved by the Ethics Review Committee of Lishui Second People’s Hospital (023/2023; approved November 17, 2023). All procedures in this study were performed in compliance with the Declaration of Helsinki. Because this study used existing clinical records, no additional intervention, prospective recruitment, or study-specific procedure was conducted for this analysis. Written informed consent for clinical data collection and future anonymized scientific use had been obtained during the original clinical data collection, and the ethics approval permitted the secondary analysis of anonymized data without additional consent.

All data used for analysis were deidentified before extraction and analysis. The analytic dataset did not contain names, contact information, medical record numbers, facial images, or other direct identifiers. Only coded and anonymized data were accessed by the research team, and the data were used only for the purposes approved by the ethics committee. No compensation was provided because no new participants were recruited and no additional study-specific procedures were performed.

All figures and supplementary materials were reviewed to ensure that no individual participant or user could be identified. For Figure 6, which shows the experimental scene, the image of the individual wearing the VR device was masked or deidentified to remove facial features and other potentially identifying information. Therefore, no individual participant or user can be identified from the revised figure, and additional consent specifically for publication of an identifiable image was not applicable.

## Results

### Acute Symptom Changes

**Figure 7.**
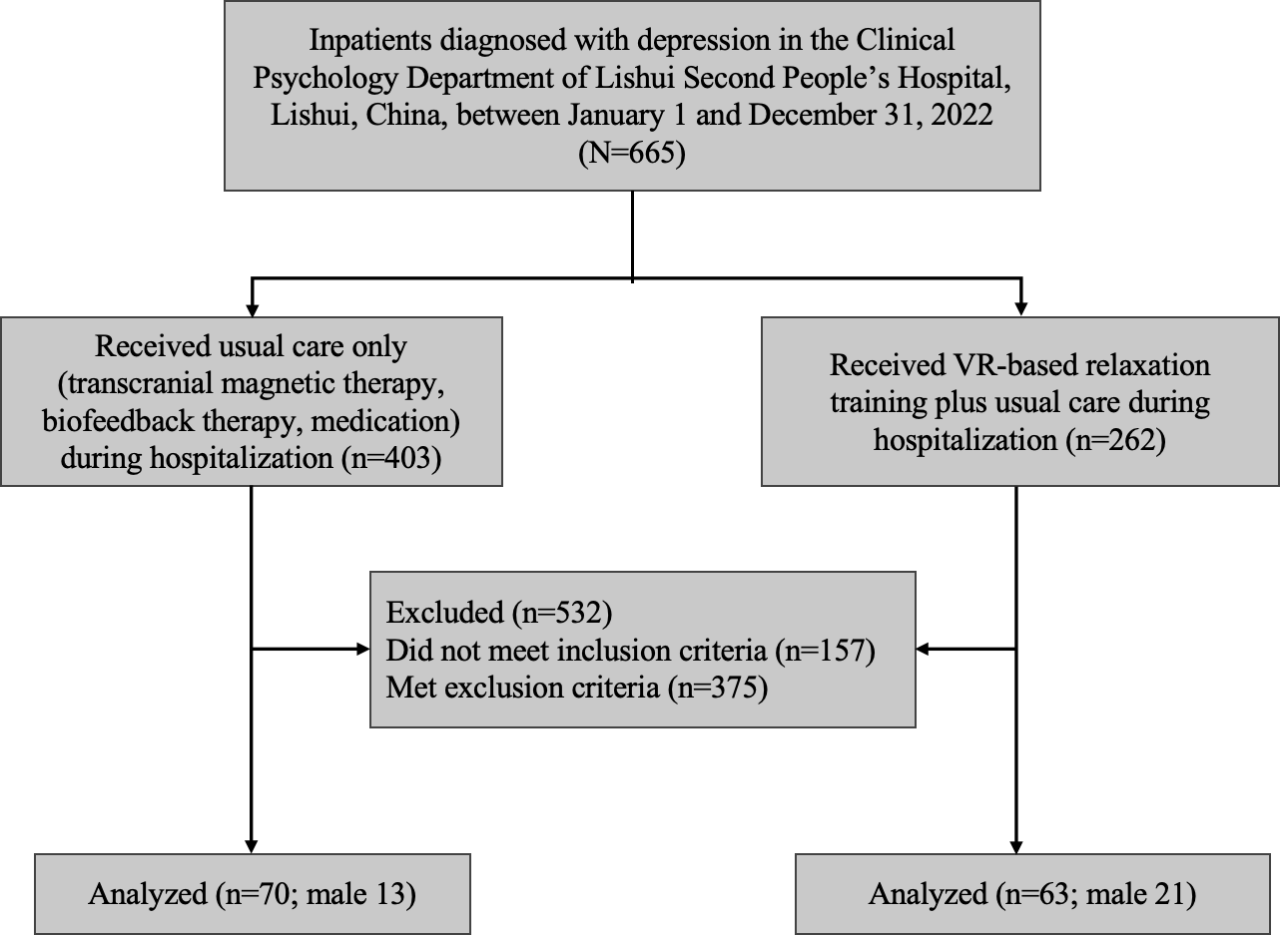
Flowchart of the procedure used to select patients maintained for the analyses. VR: virtual reality.

The patient selection flowchart is shown (Figure 7). Baseline characteristics of the study participants are presented in Table 1. There were no significant differences between the VR group and the control group in any demographic or clinical variables at baseline. The mean BMI was 21.65 (SD 3.72) in the VR group and 21.31 (SD 3.47) in the control group (mean difference 0.35, 95% CI −0.89 to 1.58; *t*_131_= 0.56; *P*=.58). The median age was 18 (IQR 15‐44) years in the VR group and 17 (IQR 15‐43) years in the control group (median difference 1.0, 95% CI −1.0 to 3.0; *U*=2010.0; *P*=.38). Similarly, no significant differences were observed in sex distribution (OR 2.19, 95% CI 0.99‐4.87; *χ*^2^_1_=3.8; *P*=.05), education level (*χ*^2^_4_=3.1; *P*=.54), marital status (*χ*^2^_2_=0.6; *P*=.73), occupation (*χ*^2^_4_=5.6; *P*=.23), or family history (OR 0.66, 95% CI 0.14‐3.07; *χ*^2^_1_=0.3; *P*=.60).

**Table 1.** Baseline characteristics and clinical outcomes by group^a^.

Variables	Virtual reality group (n=63)	Control group (n=70)	Effect size (95% CI)	Test statistic	*P* value
Continuous variables (normally distributed)^b^					
BMI (kg/m^2^), mean (SD)	21.65 (3.72)	21.31 (3.47)	0.35 (–0.89 to 1.58)	0.56 (131)	.58
Biofeedback (times), mean (SD)	9.03 (6.08)	8.60 (6.42)	0.43 (–1.72 to 2.58)	0.40 (131)	.69
Continuous variables (nonnormally distributed)^c^					
Age (y), median (IQR)	18 (15‐44)	17 (15‐43)	1.0 (–1.0 to 3.0)	2010.0	.38
Course of disease (m), median (IQR)	18 (6‐60)	12 (12‐48)	0.0 (–6.0 to 8.0)	2141.0	.77
Length of hospitalization (d), median (IQR)	14 (9‐22)	14 (9‐19)	1.0 (–2.0 to 4.0)	2050.0	.48
rTMS^d^ sessions (times), median (IQR)	5 (2-10)	5 (0‐10)	0.0 (0.0 to 3.0)	1871.0	.12
Categorical variables^e^					
Sex, n (%)			2.19 (0.99 to 4.87)	3.8 (1)	.05
Male	21 (33.3)	13 (18.6)			
Female	42 (66.7)	57 (81.4)			
Education, n (%)			—^f^	3.1 (4)	.54
Illiteracy	3 (4.8)	1 (1.4)			
Primary school	7 (11.1)	13 (18.6)			
Secondary school	19 (30.2)	24 (34.3)			
High school	25 (39.7)	23 (32.9)			
University and above	9 (14.3)	9 (12.9)			
Marital status, n (%)			—	0.6 (2)	.73
Unmarried	45 (71.4)	48 (68.6)			
Married	14 (22.2)	19 (27.1)			
Divorced	4 (6.3)	3 (4.3)			
Occupation, n (%)			—	5.6 (4)	.23
Student	39 (61.9)	43 (61.4)			
Farmer	10 (15.9)	11 (15.7)			
Self-employed or laborer	6 (9.5)	1 (1.4)			
Public institution	4 (6.3)	8 (11.4)			
Unemployed	4 (6.3)	7 (10)			
Family history, n (%)			0.66 (0.14 to 3.07)	0.3 (1)	.60
Negative	59 (93.7)	67 (95.7)			
Positive	4 (6.3)	3 (4.3)			

a Normally distributed data are represented as mean (SD) and analyzed using the independent samples *t* test; nonnormally distributed data are represented as median (IQR) and analyzed using the Mann-Whitney *U* test.

b Effect size is presented as mean difference (95% CI); test statistic is presented as *t* (*df*).

c Effect size is presented as median difference (95% CI); test statistic is presented as Mann-Whitney *U*.

d rTMS: repetitive transcranial magnetic stimulation.

e Effect size is presented as odds ratio (95% CI); test statistic is presented as *χ*2 (*df*).

f Not applicable.

Table 2 summarizes the comparisons of clinical outcomes between the 2 groups. At baseline, there were no significant differences in HAMD scores (mean difference −1.38, 95% CI −3.56 to 0.81; *t*_131_=−1.25; *P*=.22) or HAMA scores (mean difference 0.29, 95% CI −0.82 to 1.40; *t*_131_=0.52; *P*=.60) between groups.

After the intervention, the VR group showed significantly lower HAMD scores compared with the control group (mean difference −2.59, 95% CI −4.22 to −0.96; *t*_131_=−3.09; *P*=.002). Similarly, HAMA scores were significantly lower in the VR group after treatment (mean difference −2.45, 95% CI −3.64 to −1.27; *t*_131_=−4.10; *P*<.001). The 95% CIs for these differences did not cross zero, indicating a high level of precision in the estimated treatment effects.

**Table 2.** Comparison of clinical outcomes between 2 groups.

Variables	Virtual reality group (n=63)	Control group (n=70)	Effect size (95% CI)	Test statistic	*P* value
Hamilton Depression Rating Scale^a^					
Baseline score, mean (SD)	25.67 (6.29)	27.04 (6.41)	−1.38 (−3.56 to 0.81)	−1.25 (131)	.22
After treatment score, mean (SD)	7.24 (3.77)	9.83 (5.62)	−2.59 (−4.22 to −0.96)	−3.09 (131)	.002
Hamilton Anxiety Rating Scale^a^					
Baseline score, mean (SD)	17.21 (3.33)	16.91 (3.12)	0.29 (−0.82 to 1.40)	0.52 (131)	.60
After treatment score, mean (SD)	5.19 (3.19)	7.64 (3.66)	−2.45 (−3.64 to −1.27)	−4.10 (131)	<.001
Clinical response and remission outcomes^b^					
Hamilton Depression Rating Scale response (≥50% reduction), n (%)			3.72 (1.28 to 10.79)	6.4 (1)	.01
Responder	58 (92.1)	53 (75.7)			
Nonresponder	5 (7.9)	17 (24.3)			
Hamilton Depression Rating Scale remission (total score ≤7), n (%)			1.63 (0.81 to 3.24)	1.9 (1)	.17
Remission	39 (61.9)	35 (50)			
Nonremission	24 (38.1)	35 (50)			
Hamilton Anxiety Rating Scale response (≥50% reduction), n (%)			4.96 (1.87 to 13.14)	11.6 (1)	<.001
Responder	57 (90.5)	46 (65.7)			
Nonresponder	6 (9.5)	24 (34.3)			
Hamilton Anxiety Rating Scale remission (total score ≤7), n (%)			2.48 (1.16 to 5.30)	5.6 (1)	.02
Remission	49 (77.8)	41 (58.6)			
Nonremission	14 (22.2)	29 (41.4)			

a Effect size is presented as mean difference (95% CI); test statistic is presented as *t* (*df*).

b Effect size is presented as odds ratio (95% CI); test statistic is presented as *χ*2 (*df*).

For remission (total score ≤7), the VR group had a significantly higher HAMA remission rate (49/63, 77.8% vs 41/70, 58.6%; OR 2.48, 95% CI 1.16‐5.30; *χ*^2^_1_=5.6; *P*=.02). Although the point estimate suggests a clinically meaningful benefit, the CI, while not crossing 1, is relatively wide (1.16‐5.30), indicating moderate precision. In contrast, the difference in HAMD remission rates did not reach statistical significance (39/63, 61.9% vs 35/70, 50%; OR 1.63, 95% CI 0.81‐3.24; *χ*^2^_1_=1.9; *P*=.17). The CI for HAMD remission includes 1, confirming the lack of statistical significance, and its width (0.81‐3.24) reflects the uncertainty surrounding this estimate.

The HAMD response rate (≥50% reduction) was significantly higher in the VR group (58/62, 92.1% vs 53/70, 75.7%; OR 3.72, 95% CI 1.28‐10.79; *χ*^2^_1_=6.4; *P*=.01). The HAMA response rate was also significantly higher in the VR group (57/63, 90.5% vs 46/70, 65.7%; OR 4.96, 95% CI 1.87‐13.14; *χ*^2^_1_=11.6; *P*<.001). While both ORs demonstrate a substantial benefit for the VR group, the relatively wide CIs (eg, 1.28‐10.79 for HAMD response) suggest some imprecision in the exact magnitude of the effect.

### Association Between VR Relaxation Training Session Frequency and Symptom Outcomes

To further explore whether symptom outcomes varied by session frequency of VR relaxation training, the VR group was divided according to the number of treatment sessions: 2 to 4 sessions (VR1; n=19), 5 to 7 sessions (VR2; n=22), and 8 sessions or more (VR3; n=22). The VR group was also matched with control groups according to the length of hospitalization, with less than 9 days for control group 1 (CG1; n=23), 10 to 16 days for control group 2 (CG2; n=22), and 17 days or more for control group 3 (CG3; n=25).

There were no differences between the 3 treatment groups and their controls, in terms of age, course of disease, length of hospitalization, BMI, number of physiotherapy sessions (ie, biofeedback and rTMS), marital status, level of education, occupation, sex, and family history (Table S1 in Multimedia Appendix 1).

**Figure 8.**
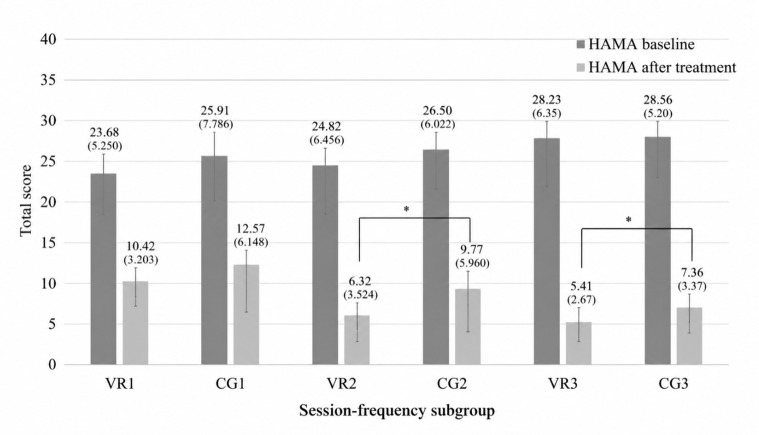
Comparison of Hamilton Depression Rating Scale (HAMD) scores at baseline and after the treatment between VR treatment and control groups (values represent mean and SD). CG1: control group 1 (length of hospitalization <9 d); CG2: control group 2 (length of hospitalization 10-16 d); CG3: control group 3 (length of hospitalization ≥17 d); VR1: virtual reality group receiving 2-4 treatment sessions; VR2: virtual reality group receiving 5-7 treatment sessions; VR3: virtual reality group receiving ≥8 treatment sessions. *P<.05, **P<.01.

At baseline, there were no significant differences in HAMD and HAMA scores between the VR groups and their controls. There were no significant differences in HAMD (*F*_5,127_=2.002;* P*=.08) and HAMA (*F*_5,127_=1.458; *P*=.21) scores among the 6 groups. At discharge, VR1’s HAMD score was not significantly different from its control group and its HAMA score was significantly lower than that of CG1 (*t(40)*=−2.338; *P*=.02). VR2’s HAMD and HAMA scores were significantly lower than those of its control group (HAMD: *t(42)*=−2.340; *P*=.02; HAMA: *t(42)*=−2.177; *P*=.04). VR3 also had significantly lower HAMD and HAMA scores than its controls (HAMD: *t(45)*=−2.645; *P*=.01; HAMA: *t(45)*=−2.969; *P*=.005; Table S2 in Multimedia Appendix 1 and Figures8  9).

There was no significant difference between VR1 and CG1 in terms of response and remission rates for depressive symptoms. VR1 had a significantly higher response rate than CG1 (*χ*²_1_=6.3; *P*=.01), whereas the remission rate did not differ significantly between groups (*χ*²_1_=3.1; *P*=.08). VR2 and CG2 did not have a significant difference in terms of response and remission rates, and the remission rate of anxiety symptoms was not significantly different; however, the response rate of anxiety symptoms was significantly higher in VR2 than in CG2 (*χ*²_1_=8.3; *P*=.004). There was no significant difference between VR3 and CG3 in the response and remission rates of both depressive and anxiety symptoms (Table S2 in Multimedia Appendix 1).

**Figure 9.**
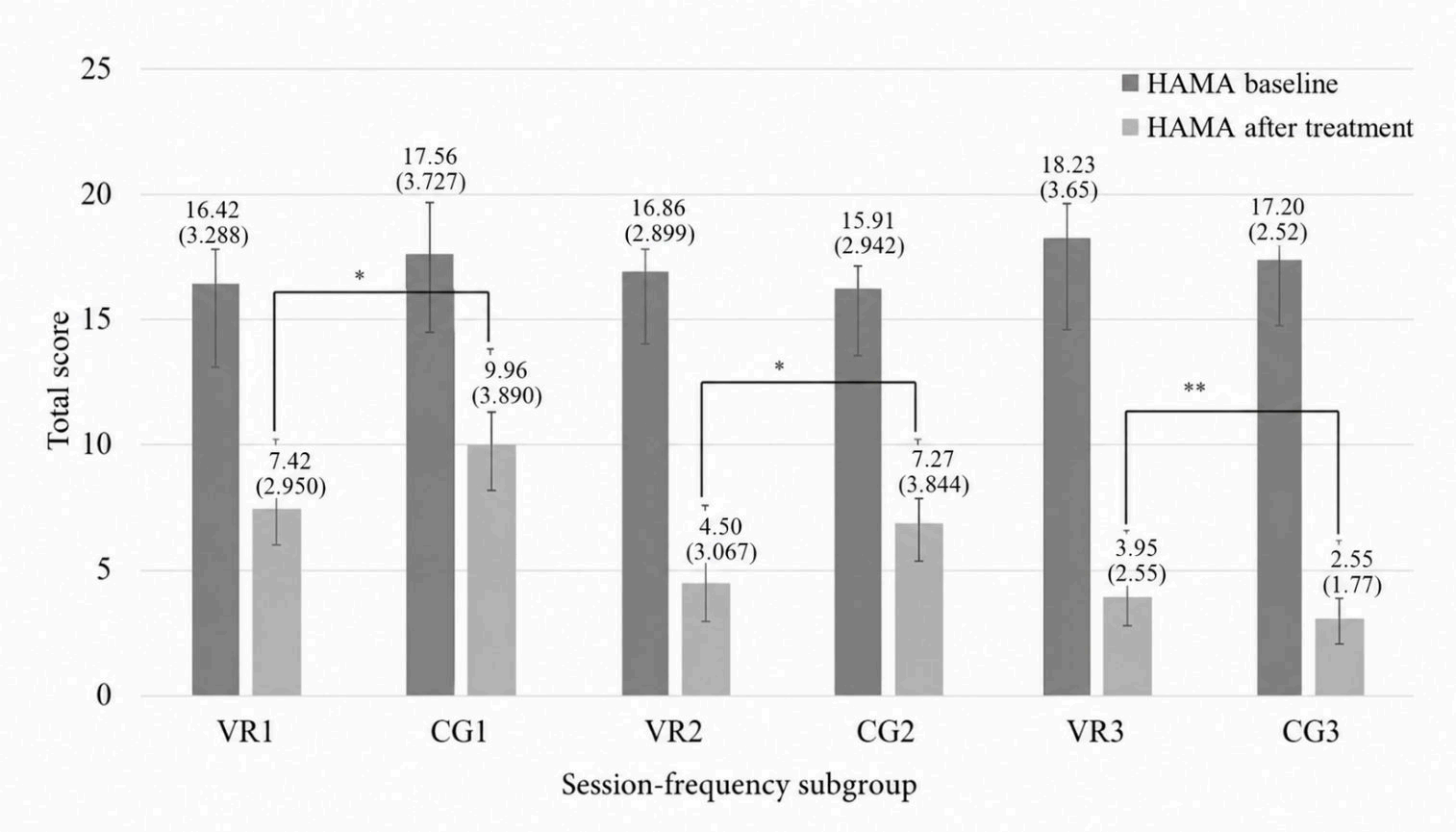
Comparison of Hamilton Anxiety Rating Scale (HAMA) scores at baseline and after the treatment between VR treatment and control groups (values represent mean and SD). CG1: control group 1 (length of hospitalization <9 d); CG2: control group 2 (length of hospitalization 10-16 d); CG3: control group 3 (length of hospitalization ≥17 d); VR1: virtual reality group receiving 2-4 treatment sessions; VR2: virtual reality group receiving 5-7 treatment sessions; VR3: virtual reality group receiving ≥8 treatment sessions. *P<.05, **P<.01.

## Discussion

### Summary of Main Findings

This retrospective study examined whether adjunctive VR-based relaxation training was associated with acute changes in depressive and anxiety symptoms among hospitalized patients with depressive disorders and explored whether symptom improvement differed by session frequency. Consistent with the study objective, patients who received adjunctive VR-based relaxation training showed greater improvement in depressive and anxiety symptoms than patients who received usual care alone. The pattern of findings was more consistent for anxiety-related outcomes than for depression remission. Exploratory session-frequency analyses suggested that anxiety improvement may be observable across relatively short VR exposure, whereas depressive symptom outcomes did not show a stable or clearly interpretable session-frequency pattern.

### Interpretation and Comparison With Existing Literature

These findings are broadly consistent with previous research suggesting that VR-supported relaxation and immersive restorative environments may be associated with reductions in anxiety, stress, and depressive symptoms [13-16,22-24]. Immersive VR relaxation may support symptom improvement through several complementary processes. Slow-paced breathing with immersive VR nature scenery has been used in heart rate variability biofeedback to facilitate relaxation-related physiological regulation [13]. Restorative VR environments have also been associated with emotional and cognitive recovery in individuals with mild-to-moderate anxiety and depression [22]. In addition, a randomized controlled trial in older women found greater improvements in depression- and anxiety-related outcomes after an immersive VR program incorporating relaxation and psychotherapeutic elements than after the comparison intervention [16]. A pilot randomized controlled trial also found that personalized VR scenarios combined with progressive muscle relaxation training were associated with greater engagement and reductions in anxiety than guided imagery [23]. These studies provide a plausible rationale for using immersive relaxation environments as an adjunctive component of care. However, the psychological and physiological mechanisms were not directly tested in this study.

The more consistent pattern observed for anxiety-related outcomes may be clinically relevant. Anxiety symptoms are common among patients with depressive disorders and are associated with greater symptom burden, functional impairment, suicide-related risk, and poorer outcomes [4-7]. One possible interpretation is that the relaxation-oriented content of the VR sessions may be particularly relevant when anxiety relief is an important clinical goal. However, this interpretation remains tentative because this study was retrospective and nonrandomized and did not isolate the effects of the immersive environment, relaxation content, or concurrent treatments.

### Exploratory Session-Frequency Patterns

The exploratory session-frequency analyses suggested that symptom outcomes, particularly anxiety-related outcomes, may vary across different levels of VR exposure. However, the pattern was not strictly linear. Because session frequency was not randomly assigned, it may have been related to hospitalization duration, clinical scheduling, patient availability, adherence, exposure to concurrent treatments, or other unmeasured factors. Accordingly, these findings should be regarded as hypothesis-generating associations rather than evidence of a causal dose-response relationship [25,26].

Multiple sessions may provide repeated opportunities to practice relaxation and engage with calming virtual environments. Previous studies have described and evaluated VR-supported progressive muscle relaxation protocols [23]. Nevertheless, the current design cannot determine whether increasing the number of sessions independently improves symptom outcomes. Future prospective studies with randomized or protocol-defined session schedules, standardized background treatments, adherence measures, and follow-up assessments are needed to clarify clinically useful session-frequency ranges and determine whether repeated VR relaxation sessions provide incremental benefits beyond usual inpatient care.

### Clinical and Real-World Implications

From a clinical perspective, adjunctive VR-based relaxation training may be considered a structured supplement to usual inpatient care rather than a replacement for established treatments. Previous studies have examined VR-supported relaxation and restorative VR environments across mental health and symptom-management contexts [13-16,22,23]. In this study, VR sessions were brief, structured, and delivered alongside routine treatment, providing preliminary real-world information about implementation in one psychiatric inpatient setting.

These findings may be relevant to hospitals interested in evaluating immersive relaxation as part of comprehensive care. However, this study did not formally assess feasibility, acceptability, staffing requirements, costs, or cost-effectiveness. These implementation outcomes, together with follow-up symptom outcomes and standardized background treatments, should be evaluated in prospective studies before broader clinical recommendations are made.

### Limitations

Several limitations should be considered. First, the retrospective, nonrandomized design prevents causal inference, and unmeasured confounding cannot be excluded, which is an important interpretive limitation of observational clinical research [25,26]. Second, the VR and control groups were naturally formed in routine care; therefore, patient preference, clinician judgment, symptom severity not captured in the records, and ward-level scheduling may have influenced group membership. Third, all patients received multiple concurrent treatments, including pharmacotherapy, rTMS, and biofeedback, and the medication type, dosage, adherence, and intensity of concomitant therapies could not be fully standardized. Fourth, the study was conducted in a single hospital and focused on acute inpatient outcomes at discharge, limiting generalizability and preventing conclusions about long-term durability. Fifth, physiological measures such as heart rate variability or cortisol were not available, so potential relaxation mechanisms could not be directly tested. Finally, the exploratory session-frequency analysis was not randomized and should be used only to generate hypotheses for future trials rather than to establish an optimal treatment frequency [25,26].

### Conclusions

This retrospective study found that adjunctive VR-based relaxation training was associated with improvements in depressive and anxiety symptoms among inpatients with depressive disorders, with a more evident pattern observed for anxiety-related outcomes. Exploratory session-frequency analyses suggested potentially relevant patterns across different levels of VR exposure, but these findings should not be interpreted as evidence of a causal dose-response effect. The study is innovative in examining structured VR-based relaxation training as an adjunct to routine care in a real-world Chinese psychiatric inpatient setting, which differs from many previous VR studies conducted in noninpatient or experimental samples [14-16,18,22,23]. The findings contribute preliminary clinical evidence regarding the integration of immersive relaxation into comprehensive inpatient care, particularly when additional anxiety relief is desired. Because the study was retrospective and nonrandomized, prospective randomized controlled trials with standardized protocols, implementation measures, and longer follow-up periods are needed to confirm these associations and clarify clinically useful session-frequency ranges.

## Supplementary material

10.2196/75251 Multimedia Appendix 1 Response and remission rates for depressive and anxiety symptoms in the virtual reality session-frequency subgroups and matched control subgroups.

## Abbreviations

HAMA: Hamilton Anxiety Rating Scale
HAMD: Hamilton Depression Rating Scale
*ICD-10*: *International Classification of Diseases, Tenth Revision*
MDD: major depressive disorder
OR: odds ratio
rTMS: repetitive transcranial magnetic stimulation
VR: virtual reality
WHO: World Health Organization
