# Supplementary material for: Virtual Reality–Based Relaxation Training and Symptom Improvement Among Inpatients With Depressive Disorders: Retrospective Nonrandomized Comparative Study
Source: JMIR Form Res. 2026 Jul 3;10:e75251. doi: 10.2196/75251 (PMC13331400; doi:10.2196/75251)
Supplement: Multimedia Appendix 1 [file formative-v10-e75251-s001.docx]

**Table 1**. Demographic and clinical characteristics of participants at baseline

|  | VR1（n = 19） | VR2（n = 22） | VR3（n = 22） | CG1（n = 23） | CG2（n = 22） | CG3（n = 25） | （VR1）VS（CG1） | （VR2）VS（CG2） | （VR3）VS（CG3） |
| --- | --- | --- | --- | --- | --- | --- | --- | --- | --- |
|  | *M*（SD） | *M*（SD） | *M*（SD） | *M*（SD） | *M*（SD） | *M*（SD） | *t*（*P*） | *t*（*P*） | *t*（*P*） |
| Age, years | 27.32（15.91） | 28.63（16.58） | 25.64（17.10） | 26.48（16.14） | 27.36（16.80） | 25.88（18.25） | 0.16（ .867） | 0.199（ .843） | -0.047（ .963） |
| Course of disease, month | 30.18（37.18） | 45.95（50.61） | 30.55（33.12） | 39.17（66.65） | 28.45（28.42） | 40.80（54.43） | -0.524（ .603） | 1.414（ .165） | -0.767（ .447） |
| Length of hospitalization, days | 6.89（1.66） | 14.00（1.98） | 25.36（5.71） | 7.09（1.81） | 13.00（1.97） | 22.92（4.20） | -0.356（ .724） | 1.678（ .101） | 1.395（ .170） |
| BMI | 22.54（4.440） | 21.67（3.92） | 20.86（2.70） | 20.77（2.480） | 20.71（3.31） | 22.33（4.20） | 1.632（ .110） | 0.886（ .381） | -1.403（ .168） |
| Biofeedback, times | 4.63（1.422） | 8.00（4.11） | 13.86（6.89） | 3.78（2.044） | 7.50  （3.65） | 14.52（6.02） | 1.529（ .134） | 0.427（ .672） | -0.349（ .729） |
| rTMS, times | 3.11（2.424） | 6.00（4.22） | 7.95（3.39） | 2.26（2.816） | 6.00（4.07） | 6.00（3.91） | 1.029（ .310） | 0.000（1.000） | 1.821（ .075） |
|  | *N*（%） | *N*（%） | *N*（%） | *N*（%） | N（%） | N（%） | χ2（*P*） | χ2（*P*） | χ2（*P*） |
| Gender |  |  |  |  |  |  | 0.521（ .470） | 0.983（ .322） | 2.744（ .098） |
| Male | 6（31.6） | 8（36.4） | 7（31.8） | 5（21.7） | 5（22.7） | 3（12.0） |  |  |  |
| Female | 13（68.4） | 14（63.6） | 15（68.2） | 18（78.3） | 17（77.3） | 22（88.0） |  |  |  |
| Education |  |  |  |  |  |  | 1.098（ .895） | 3.867（ .424） | 2.300（ .681） |
| Illiteracy | 0（0） | 2（9.1） | 1（4.5） | 1（4.3） | 0（0） | 0（0） |  |  |  |
| Primary school | 1（5.3） | 3（13.6） | 3（13.6） | 2（8.7） | 7（31.8） | 4（16.0） |  |  |  |
| Secondary school | 6（31.6） | 6（27.3） | 7（31.8） | 7（30.4） | 6（27.3） | 11（44.0） |  |  |  |
| High school | 8（42.1） | 8（36.4） | 9（40.9） | 9（39.1） | 7（31.8） | 7（28.0） |  |  |  |
| University and above | 4（21.1） | 3（6.1） | 2（9.1） | 4（17.4） | 2（9.1） | 3（12.0） |  |  |  |
| Marriage |  |  |  |  |  |  | 1.050（ .592） | 2.727（ .256） | 1.662（ .436） |
| Unmarried | 13（68.4） | 15（68.2） | 17（77.3） | 16（69.6） | 14（69.7） | 18（72.0） |  |  |  |
| Married | 5（26.3） | 5（22.7） | 4（18.2） | 4（17.4） | 8（27.3） | 7（28.0） |  |  |  |
| Divorcee | 1（5.3） | 2（9.1） | 1（4.5） | 3（13.0） | 0（0） | 0（0） |  |  |  |
| Profession |  |  |  |  |  |  | 5.056（ .282） | 1.444（ .836） | 7.010（ .135） |
| Student | 10（52.6） | 13（59.1） | 16（72.7） | 13（54.8） | 13（59.1） | 17（68.0） |  |  |  |
| Farmer | 1（5.3） | 5（22.7） | 4（18.2） | 4（17.4） | 4（18.2） | 3（12.0） |  |  |  |
| Self-employed or laborer | 3（15.8） | 1（4.5） | 2（9.1） | 0（0） | 1（4.5） | 0（0） |  |  |  |
| Public institution | 3（15.5） | 1（4.5） | 0（0） | 3（13.0） | 3（13.6） | 2（8.0） |  |  |  |
| Unemployed | 2（10.5） | 2（9.1） | 0（0） | 3（13.0） | 1（4.5） | 3（12.0） |  |  |  |
| Family history |  |  |  |  |  |  | 1.240（ .265） | 2.095（ .148） | 1.396（ .237） |
| Negative | 18（94.7） | 22（100） | 19（86.4） | 23（100） | 20（90.9） | 24（96.0） |  |  |  |
| Positive | 1（5.3） | 0（0） | 3（13.6） | 0（0） | 3（9.1） | 1（3.8） |  |  |  |

**Table 2** Comparison of response and remission rates among the groups

|  | VR1(n = 19) | VR2(n = 22) | VR3(n = 24) | CG1(n = 23) | CG2(n = 22) | CG3(n = 25) | (VR1)VS(CG1) | (VR2)VS(CG2) | (VR3)VS(CG3) |
| --- | --- | --- | --- | --- | --- | --- | --- | --- | --- |
|  | *M*(SD) | *M*(SD) | *M*(SD) | *M*(SD) | *M*(SD) | *M*(SD) | *t*(*P*) | *t*(*P*) | *t*(*P*) |
| HAMD |  |  |  |  |  |  |  |  |  |
| Baseline | 23.68(5.250) | 24.82(6.456) | 28.23(6.35) | 25.91(7.786) | 26.50(6.022) | 28.56(5.20) | -1.063 ( .294) | -0.894(0.377) | -0.198( .844) |
| Post-treatment | 10.42(3.203) | 6.32(3.524) | 5.41(2.67) | 12.57(6.148) | 9.77(5.960) | 7.36(3.37) | -1.372 ( .178) | -2.340^*^(0.024) | -2.177^*^( .035) |
| HAMA |  |  |  |  |  |  |  |  |  |
| Baseline | 16.42(3.288) | 16.86(2.899) | 18.23(3.65) | 17.56(3.727) | 15.91(2.942) | 17.20(2.52) | -1.044 ( .303) | 1.084(0.285) | 1.134( .263) |
| Post-treatment | 7.42(2.950) | 4.50(3.067) | 3.95(2.55) | 9.96(3.890) | 7.27(3.844) | 2.55(1.77) | -2.338^*^( .024) | -2.645^*^( .011) | -2.969^**^( .005) |
|  | *N*(%) | *N*(%) | *N*(%) | *N*(%) | N(%) | N(%) | *χ*2(*P*) | *χ*2(*P*) | *χ*2(*P*) |
| HAMD response |  |  |  |  |  |  | 3.249( .071) | 3.088( .079) | 0.899( .343) |
| HAMD reduce rate ≥50% | 15(78.9) | 21(95.5) | 22(100) | 12(52.2) | 17(77.3) | 24(96.0) |  |  |  |
| HAMD reduce rate＜50% | 4(21.1) | 1(4.5) | 0(0) | 11(47.8) | 5(22.7) | 1(4.0) |  |  |  |
| HAMD remission |  |  |  |  |  |  | 0.492( .483) | 0.863( .353) | 0.812( .368) |
| HAMD total score ≤7 | 5(26.3) | 15(68.2) | 19(86.4) | 4(17.4) | 12(54.5) | 19(76.0) |  |  |  |
| HAMD total score＞7 | 14(73.7) | 7(31.8) | 3(13.6) | 19(82.6) | 10(45.5) | 6(24.0) |  |  |  |
| HAMA response |  |  |  |  |  |  | 6.313^*^( .012) | 8.324^**^( .004) | 0.234( .629) |
| HAMA reduce rate ≥50% | 14(73.7) | 22(100) | 21(95.5) | 8(34.8) | 15(68.2) | 23(92.0) |  |  |  |
| HAMA reduce rate＜50% | 5(26.3) | 0(0) | 1(4.5) | 15(65.2) | 7(31.8) | 2(8.0) |  |  |  |
| HAMA remission |  |  |  |  |  |  | 3.109( .078) | 3.030( .082) | 0.502( .479) |
| HAMA total score ≤7 | 10(52.6) | 19(86.4) | 20(90.9) | 6(26.1) | 14(63.6) | 21(84.0) |  |  |  |
| HAMA total score＞7 | 9(47.4) | 3(13.6) | 2(9.1) | 17(73.9) | 8(36.4) | 4(16.0) |  |  |  |

Note. * *P* < 0.05，** *P* < 0.01.
